# The Infected District

**Published:** 1873-08-15

**Authors:** 


The Infected District. — The locality where the cases of cholera reported at the last meeting of the Society of Physicians and Surgeons have occurred, is a new district, lately settled, where the open prairie has only the past season been turned up, roads cut through, etc. No sewers are yet put down, and the lake water has been introduced only during the last few weeks, since the outbreak of the epidemic. The district is in a measure isolated from the more thickly settled districts of the city, and it has not, apparently, as yet spread much beyond the limits there mentioned. Isolated sporadic cases, however, have occurred in many of the more crowded, filthy districts in various parts of the city. The family in which the disease first broke out at 945 Butterfield street had been living here in the city for two years, and it cannot be learned that they had been in contact with any emigrants or recently-arrived strangers.

